# Coverage of insecticide-treated mosquito nets in preschool consultations and factors associated with non-use of insecticide-treated mosquito nets among children in the Democratic Republic of Congo: A base population cross-sectional study

**DOI:** 10.1371/journal.pone.0345190

**Published:** 2026-05-22

**Authors:** Aliocha Natuhoyila Nkodila, Stanislas Okitasho Wembomyama, Reagan Okingo Luvande, André-Ronz Basilua Nsana, Philippe Ngwala Lukanu

**Affiliations:** 1 Department of Family Medicine and Primary Health Care, Protestant University in Congo, Kinshasa, Democratic Republic of the Congo; 2 Rural Health Care Association (ASBL), Kinshasa, Democratic Republic of the Congo; 3 School of Public Health, University of Goma, Goma, Democratic Republic of the Congo; 4 National Mental Health Program, Ministry of Public Health, Kinshasa, Democratic Republic of the Congo; Department of Medical Research (Lower Myanmar) Advanced Molecular Research Center, MYANMAR

## Abstract

Insecticide-treated nets (ITNs) are among the most effective malaria prevention interventions, yet their actual use among children remains below national targets. This study assesses ITN coverage and utilization among children attending preschool consultations (PSC) and identifies factors associated with non-use. A cross-sectional study was conducted in 2025 in 156 health facilities across six provinces of the Democratic Republic of the Congo (DRC). Children aged 6–59 months accompanied by a parent or guardian were included. Data were collected during a household survey through face-to-face interviews using a structured questionnaire programmed in ODK Collect. Data on ITN ownership and use, as well as sociodemographic characteristics, were collected. Factors associated with non-use were analyzed using multivariate logistic regression. Among the 1,247 children included in preschool consultations, the mean age was 15.0 ± 4.3 months. The population showed marked socioeconomic vulnerability: 81.5% of guardians had a low educational level, 82.4% of households had low monthly income, and 48.2% lived in rudimentary housing. Community awareness through community health workers (CHWs) was high (78.7%). Overall, 62.5% of households owned at least one ITN, and 95.9% of children used it. The main factors associated with non-use were lack of community sensitization (aOR = 4.75), parental marital status (aOR = 4.57), rainy season (aOR = 3.41), rudimentary housing (aOR = 2.59), and a high number of children in the household (aOR = 1.80). Adherence to ITN use was ≥ 80% of individuals reporting sleeping under an ITNs the previous night, a predefined threshold based on WHO recommendations. Community-based interventions and context-adapted planning that account for seasonal and socioeconomic factors are essential to maximize the impact of malaria reduction among preschool children in the DRC.

## Introduction

Malaria remains a major public health problem in the Democratic Republic of the Congo (DRC), where it is one of the leading causes of morbidity and mortality among children under five years of age [1,2]. The DRC is among the countries bearing the heaviest global malaria burden, contributing substantially to the overall disease burden in sub-Saharan Africa [3]. In this context, vector control is a crucial strategy for reducing malaria incidence, particularly among vulnerable populations such as preschool-aged children [4–6]. Insecticide-treated nets (ITNs) are recognized by the World Health Organization (WHO) as one of the most effective preventive interventions against bites from *Anopheles* mosquitoes, the vectors responsible for malaria transmission [7]. The effectiveness of ITNs relies on two complementary mechanisms: physical protection against mosquito bites and insecticidal action that reduces vector density [8,9]. Numerous studies have demonstrated a significant reduction in malaria incidence and child mortality in areas where ITN use is high [7,10]. In the DRC, several free ITN distribution campaigns have been implemented as part of national malaria control programs. Recent data from the Demographic and Health Survey (DHS-RDC III, 2023–2024) indicate relatively high household net ownership, estimated at approximately 69% in some provinces [11]. However, effective use of these nets among children under five remains below national targets, defined as ≥80% coverage, with only about 57% of children reported to have slept under an ITN the night before the survey [11]. This discrepancy between ownership and actual use is a major concern, as it limits the expected impact of preventive interventions on reducing the malaria burden. Determinants of ITN use are multifactorial and vary according to sociocultural and economic contexts. Studies conducted in various African countries have identified factors such as parental education level, knowledge of malaria transmission, perception of net effectiveness, availability of sufficient nets for all household members, and practical barriers such as discomfort due to heat or difficulty in installation [12,13]. Children under five years of age represent a biologically and epidemiologically distinct high-risk group for malaria, with significantly higher morbidity and mortality compared to older children and adults. For this reason, national malaria control strategies in the DRC and WHO guidelines prioritize this age group for preventive interventions, including universal access and use of insecticide-treated nets (ITNs) [13]. Furthermore, evidence from the literature suggests that ITN use is not uniformly distributed within households, and that children under five are often prioritized for net use due to their heightened vulnerability [14]. However, intra-household allocation may still be influenced by factors such as household net availability, caregiver knowledge, education level, perception of malaria risk, and cultural or practical sleeping arrangements. Given this epidemiological vulnerability and programmatic prioritization, focusing the analysis on children under five allows for a more targeted assessment of equity and effectiveness in the implementation of malaria prevention strategies [15].

In the DRC, despite the importance of these factors, few studies have comprehensively explored the individual and contextual determinants explaining why some children do not consistently sleep under an ITN. A thorough understanding of factors associated with non-use is essential to guide targeted intervention strategies, improve community adherence, and maximize the impact of malaria control programs. This is particularly critical in the DRC, where health systems face logistical challenges, resource constraints, and heterogeneous local contexts. This study aims to assess the coverage and utilization rate of insecticide-treated nets among children attending preschool consultations in the DRC and to identify individual, social, and contextual factors associated with ITN non-use.

## Methods

### Study design and population

This analytical cross-sectional study was conducted from July 1 to August 30, 2025, which represents the complete period of data collection, in 156 health facilities providing preschool consultations (PSC) across six provinces of the Democratic Republic of the Congo (DRC). In the six study provinces, 1,560 health facilities providing PSC were identified; however, due to resource constraints, a sample of 10% of these facilities was selected using simple random sampling. PSC services represent a key point of contact for monitoring the health of children under five years of age, including vaccination, malnutrition screening, and promotion of malaria preventive interventions. The target population consisted of children aged 6–59 months accompanied by a parent or legal guardian who consented to participate in the study. Children requiring immediate hospitalization, those who received the ITNs for both reasons, or whose parent refused participation were excluded. A multistage sampling procedure was applied. First, representative provinces were selected based on their epidemiological profile and reported mosquito net coverage from DHS2 data. Second, health facilities offering PSC were randomly selected within each province. Finally, children were systematically selected according to a predefined sampling interval as they attended consultations. The sample size was calculated using the expected proportion of children using insecticide-treated nets (57%) [11], with a 95% confidence level, a 5% margin of error, and an anticipated 10% non-response rate. The calculated minimum sample size was equal to or greater than 415 mother–child pairs.

### Data collection procedure

Data were collected using a structured questionnaire administered to parents or guardians and complemented, whenever possible, by direct observation of mosquito nets available in the household. Each parent or guardian was required to describe how they had obtained the insecticide-treated net (ITN) during PSC. All information included in this study was obtained through interviews conducted with parents of children in the households. Collected information included sociodemographic variables (guardian’s age, educational level, parents’ occupation, parents’ income level, number of children, type of housing, and child’s age), ITN-related variables (ownership, reasons for non-ownership, number of nets, use the night preceding the survey, reasons for ITN use, and sensitization regarding ITN use). The duration since ITN acquisition was obtained during the interview. Parents or guardians were asked to recall the month in which they received the ITN during antenatal care (ANC) or PSC. ITN coverage was defined as the proportion of households owning at least one insecticide-treated net received during PSC, while the utilization rate was defined as the proportion of children who slept under an ITN the night preceding the survey among those who had received an ITN. In other words, the following definitions were considered:

- ITN ownership refers to the proportion of households reporting possession of at least one insecticide-treated net.
- ITN utilization refers to the proportion of children under five who slept under an ITN the night preceding the survey.
- ITN coverage has been clarified in the manuscript as referring to access and/or availability indicators depending on the context of analysis, and is now consistently defined throughout the Methods, Results, and figure captions.

In the context of the study conducted in the Democratic Republic of the Congo, the rainy season is generally associated with higher malaria transmission and increased mosquito density, which can influence both the distribution and use of insecticide-treated bed nets (ITNs). Therefore, we compared ITN acquisition across seasons to assess potential seasonal variations in distribution and access. For the purposes of this study, the rainy season was defined as the period from September to April, and the dry season as the remaining months of the year.

### Statistical analysis

Data were entered and analyzed using SPSS version 25. Qualitative variables were summarized using frequencies and percentages, and quantitative variables were presented as means and standard deviations. Associations between ITN non-use and potentially related factors were assessed using the chi-square test for categorical variables, Student’s *t*-test for normally distributed continuous variables, and the Mann–Whitney U test for non-normally distributed continuous variables. Univariate and multivariate logistic regression analyses were performed to identify factors independently associated with ITN non-use, using a stepwise approach. Only variables that were statistically significant in univariate analysis were included in the multivariate model. Results were expressed as adjusted odds ratios (aOR) with 95% confidence intervals (CI) to measure the strength of association between independent variables and ITN non-use. A p-value < 0.05 was considered statistically significant. The study was submitted to and approved by the National Health Ethics Committee of the DRC under reference number n°259/CNS/BN/PMMF/2023. Written informed consent was obtained from parents or legal guardians, and all data were anonymized to ensure participant confidentiality. We did not have access to any information identifying individual participants during or after data collection.

## Results

In this study, we included 1,247 households in which a parent or guardian was interviewed. The results in this table were calculated based on the sample size. Table 1 shows that children attending preschool consultations had a mean age of 15.0 ± 4.3 months, and their guardians were relatively young (28.4 ± 6.6 years). The majority of parents were living with a partner (77.4%). The overall socioeconomic level appeared low: 81.5% of guardians had a low educational level, and 82.4% of households had a low monthly income (median: USD 40). More than half of the mothers were homemakers (57.7%). The median household size was two children. Regarding housing conditions, 48.2% lived in rudimentary dwellings. Community sensitization through community health workers (CHWs) was high (78.7%). Finally, ITN acquisition was evenly distributed between the dry season (50.8%) and the rainy season (49.2%). Overall, the study population was characterized by marked socioeconomic vulnerability, alongside high coverage of community sensitization (Table 1).

**Table 1:** General characteristics of guardians and children attending preschool consultations.

Variable	Frequency (n = 1247)	Percentage (%)
Mean age of children (months)	15.0 ± 4.3	
Mean age of guardians (years)	28.4 ± 6.6	
Parental marital status		
Living with partner	965	77.4
Living without partner	282	22.6
Education level		
Low level	1016	81.5
High level	231	18.5
Mother’s occupation		
Homemaker	720	57.7
Employed outside the home	527	42.3
Monthly household income (USD)	40.0 (4.8–120.0)	
Low income	1028	82.4
High income	219	17.6
Median number of children in household	2.0 (1.0–3.0)	
Type of housing		
Rudimentary house	601	48.2
Permanent material house	646	51.8
Community sensitization by CHW on ITN use		
No	266	21.3
Yes	981	78.7
Season of ITN acquisition		
Rainy season	614	49.2
Dry season	633	50.8

Table 2 shows that nearly two-thirds of households owned at least one insecticide-treated net (ITN) (62.5%). Among those who did not own an ITN, the main reason reported was lack of information (51.5%), followed by stock-outs at health centers (35.3%). Among households that owned an ITN, its use was very high (95.9%). The primary reason for use was protection of the child against malaria (69.1%), followed by prevention of mosquito bites (28.1%). Other motivations were marginal. Overall, these results indicate that non-ownership of ITNs is mainly related to informational and organizational barriers, whereas adherence to use is excellent when the ITN is available (Table 2).

**Table 2:** Characteristics of ITN ownership and utilization.

Variable	n	Frequency	Percentage (%)
ITN ownership	1247		
No		468	37.5
Yes		779	62.5
Reason for non-ownership of ITN	468		
Lack of information about the availability of mosquito nets and how to use them		241	51.5
ITN stock-out at health center		165	35.3
Missed appointment to collect ITN		44	9.4
Change of health area		18	3.8
ITN utilization	779		
No		32	4.1
Yes		747	95.9
Reasons for use	747		
Protect the child against malaria		516	69.1
Avoid mosquito bites		210	28.1
Recommended by health providers		14	1.9
Child completed vaccination schedule		6	0.8
Does not know		1	0.1

### ITN coverage according to household sensitization and season

**Fig 1:**
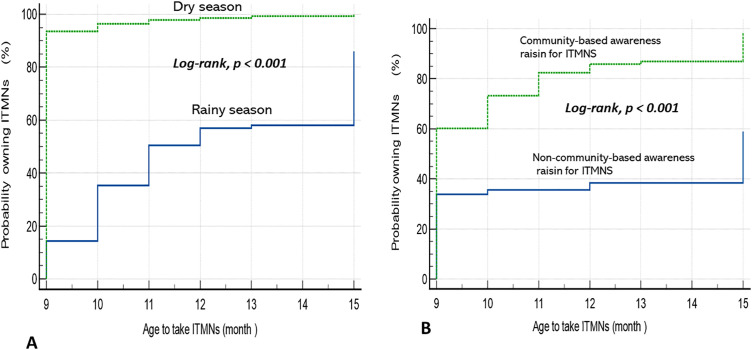
ITN coverage according to (A) household sensitization and (B) season of ITN acquisition.

Fig 1 shows a significant difference in ITN coverage according to the season of acquisition and the type of sensitization (log-rank, p < 0.001). Coverage was faster and higher when ITNs were obtained during the dry season compared to the rainy season, suggesting better accessibility or organization of distribution activities during this period (Fig 1A).

Households that benefited from community sensitization had a markedly higher and earlier probability of obtaining ITNs compared to those without sensitization, highlighting the decisive role of community-based interventions (Fig 1B).

Table 3 highlights several factors significantly associated with ITN non-use. Non-user children were older (p = 0.009) and lived in households with a higher number of children (p = 0.022). Non-use was more frequent among parents living without a partner (p < 0.001), those with a low level of education (p = 0.016), and those with low income (p = 0.009). It was also associated with rudimentary housing (p = 0.017). Community sensitization by community health workers was strongly associated with ITN use: absence of sensitization was predominant among non-users (68.8%; p < 0.001). Finally, the rainy season was significantly associated with higher non-use (p < 0.001). In contrast, mother’s age, age at ITN receipt, and occupation did not show a significant association (Table 3).

**Table 3:** General characteristics of the study population according to ITN utilization status.

Variable	ITN Non-use (n = 32)	ITN Use (n = 747)	p-value
Child’s age (months)	16.2 ± 4.9	12.8 ± 4.2	**0.009**
Age at ITN receipt (months)	9.1 ± 0.4	9.4 ± 1.1	0.241
Mother’s age (years)	27.8 ± 5.9	28.7 ± 6.6	0.460
Number of children (median, IQR)	4.0 (2.0–4.8)	2.0 (1.0–3.0)	**0.022**
Marital status			**< 0.001**
Living with partner	13 (40.6)	586 (78.4)	
Living without partner	19 (59.4)	161 (21.6)	
Monther’s education level			**0.016**
Low level	29 (90.6)	600 (80.3)	
High level	3 (9.4)	147 (19.7)	
Monther’s occupation			0.237
Homemaker	21 (65.6)	430 (57.6)	
Employed	11 (34.4)	317 (42.4)	
**Total household income**			**0.009**
Low income	30 (93.8)	625 (83.7)	
High income	2 (6.3)	122 (16.3)	
Type of housing			**0.017**
Rudimentary house	21 (65.6)	335 (44.8)	
Permanent material house	11 (34.4)	412 (55.2)	
Community sensitization on ITN use			**< 0.001**
No	22 (68.8)	74 (9.9)	
Yes	10 (31.3)	673 (90.1)	
Season of ITNs acquisition			**< 0.001**
Rainy season	25 (78.1)	153 (20.5)	
Dry season	7 (21.9)	259 (79.5)	

Multivariate analysis identified several factors independently associated with non-use of ITNs received during preschool consultations (PSC). Children from larger households had an increased risk of non-use (aOR = 1.80; p = 0.017). Similarly, living without a partner was associated with nearly a fivefold higher risk of non-use (aOR = 4.57; p < 0.001). Rudimentary housing remained significantly associated with non-use (aOR = 2.59; p = 0.031). Lack of sensitization by community health workers was a major determinant (aOR = 4.75; p < 0.001), as was the rainy season (aOR = 3.41; p < 0.001).

In contrast, child’s age, education level, and monthly income were no longer statistically significant after adjustment (Table 4).

**Table 4:** Factors associated with non-use of ITNs received during preschool consultations among children.

Variable	Univariate Analysis		Multivariate Analysis	
	p-value	OR (95% CI)	p-value	aOR (95% CI)
Child’s age (months) **	**0.007**	1.28 (1.06–1.36)	0.185	0.93 (0.85–1.03)
Number of children in household **	**0.025**	1.85 (1.38–2.98)	**0.017**	1.80 (1.36–2.96)
Parental marital status				
Living with partner		1		1
Living without partner	**<0.001**	5.32 (2.57–11.00)	**<0.001**	4.57 (2.72–8.66)
Monther’s education level				
High		1		1
Low	**0.016**	2.37 (1.12–7.88)	0.959	1.16 (0.22–4.12)
**Total household income**				
High		1		1
Low	**0.045**	1.71 (1.31–3.52)	0.291	1.66 (0.65–4.23)
Type of housing				
Permanent material house		1		1
Rudimentary house	**0.024**	2.35 (1.12–4.94)	**0.031**	2.59 (1.65–3.89)
Community sensitization on ITN use				
Yes		1		1
No	**<0.001**	5.01 (2.13–13.87)	**<0.001**	4.75 (2.93–8.44)
Season of ITNs acquisition				
Dry season		1		1
Rainy season	**<0.000**	3.87 (1.89-6.66)	**<0.001**	3.41 (1.94-7.07)

## Discussion

This study highlights a population of children attending preschool consultations who mostly come from socio-economically vulnerable households. The mean age of the children (15 months) corresponds to a period of high susceptibility to malaria, particularly in sub-Saharan Africa, where children under five remain the most affected by malaria morbidity and mortality [14,15]. The young age of the guardians (mean 28 years) reflects the demographic profile of urban households in Central Africa, characterized by early motherhood and high economic dependence [16]. The predominance of low educational level (81.5%) and low income (82.4%) confirms the significant role of social determinants in access to preventive interventions. Recent analyses in sub-Saharan Africa show that socio-economic status significantly influences ITN ownership and use, notably through access to information and health services [17,18]. However, in our study, education level and income were not significant after adjustment, suggesting that their effect may be mediated by other structural factors, particularly community sensitization and housing conditions. ITN coverage (62.5%) remains below the international universal coverage targets set by the WHO, which aim for equitable access for all at-risk populations [15]. The main barrier to non-ownership was lack of information (51.5%), followed by stock-outs (35.3%). These findings align with recent reports from the RBM Partnership to End Malaria (2022–2024), which emphasize that current challenges involve not only demand but also supply continuity and the performance of distribution systems [19,20]. In contrast, ITN use was very high (95.9%) when nets were available. The high level of ITN utilization among households that owned a net suggests strong acceptability and perceived effectiveness of mosquito nets in preventing malaria. This finding is consistent with previous studies in sub-Saharan Africa showing that when ITNs are available, their use is generally high, particularly for young children who are perceived as highly vulnerable [12,15]. However, overall ownership remains below universal coverage targets, indicating that access, not acceptability, is the primary bottleneck. The most frequently reported reason for non-ownership was lack of information (51.5%), followed by stock-outs at health facilities (35.3%). These findings suggest that both demand-side and supply-side constraints are contributing to inadequate coverage. This result is consistent with recent data indicating that once an ITN is acquired, adherence to its use is generally high, particularly when malaria risk perception is strong [21,22]. The main motivation (protecting the child from malaria) reflects good understanding of the health benefit, highlighting the effectiveness of prevention messages. Our data confirm the decisive impact of community sensitization: households exposed to community health workers (CHWs) had faster and higher ITN coverage.

The identification of “lack of information” as the leading reason for non-ownership highlights important gaps in health communication and community engagement. This may reflect insufficient awareness of distribution campaigns, unclear eligibility criteria, or limited understanding of where and when ITNs can be obtained. Similar findings have been reported in Nigeria and Uganda, where information gaps significantly reduced uptake of preventive interventions [23]. Stock-outs at health facilities further indicate weaknesses in supply chain management and distribution systems. Inconsistent availability of ITNs at service delivery points undermines universal coverage efforts and reflects systemic inefficiencies in procurement and logistics [4]. Together, these findings suggest that improving ITN coverage requires strengthening both health system readiness and community-level awareness.

Importantly, this study shows that non-use of ITNs was very low (4.1%), indicating strong adherence when nets are available. Nevertheless, multivariate analysis identified several determinants of non-use, including household structure, housing conditions, lack of community sensitization, and seasonal factors. Absence of sensitization increased the risk of non-use nearly fivefold (aOR = 4.75). This finding aligns with WHO strategic recommendations, which emphasize integrating community approaches to improve sustainable adoption of vector control interventions [15]. Community agents play a key role in reinforcing social norms that support systematic ITN use [23]. Season was also an independent factor: the rainy season was associated with increased non-use (aOR = 3.41). Although malaria transmission is higher during this period, logistical constraints (access to facilities, humidity, housing conditions) may limit effective use. Recent reports indicate that seasonal variations affect not only transmission but also the availability and use of preventive tools [15,24]. Furthermore, living without a partner significantly increased the risk of non-use (aOR = 4.57), suggesting that spousal support may facilitate adherence to health recommendations by reinforcing decision-making and household economic stability [25]. Likewise, larger households had a higher risk of non-use (aOR = 1.80), likely due to competition for available resources (insufficient number of nets relative to the number of children) [26]. Rudimentary housing remained associated with non-use (aOR = 2.59), which can be explained by structural constraints (lack of hanging points, overcrowding, excessive heat) limiting proper ITN installation [27].

The strong associations observed for variables such as sensitization and marital status may be explained by several contextual and methodological factors. Regarding sensitization, this association may reflect a true effect of health communication interventions, whereby individuals exposed to malaria prevention messages are more likely to adopt and report the use of insecticide-treated nets (ITNs). However, it may also be influenced by reverse causality, as individuals already engaged with health services—such as those attending child health consultations—are more likely to receive sensitization messages, potentially inflating the observed association. For marital status, the observed association may be explained by the presence of greater social and economic support within married households, which can facilitate both access to and consistent use of ITNs. In addition, spouses may play a role in household health decision-making, thereby reinforcing preventive health behaviors. Nevertheless, this association may also be partially confounded by unmeasured variables such as socioeconomic status, education level, or household stability, which are known to influence both marital status and health-related behaviors.

These findings have important implications for malaria control policies in the DRC. First, addressing information gaps through strengthened community health education is essential. Expanding the role of community health workers in delivering targeted messaging could significantly improve both ownership and utilization. Second, improving supply chain efficiency is critical to eliminate stock-outs at health facilities. Ensuring continuous availability of ITNs, particularly in high-risk pediatric populations, should remain a programmatic priority. Third, social and household vulnerabilities must be considered in intervention design. Targeted strategies for single-parent households, larger families, and households in poor housing conditions may help reduce inequities in ITN use. Finally, integration of seasonal planning into malaria prevention campaigns could improve timing and effectiveness of distribution and behavior change interventions.

This study has several limitations. First, the cross-sectional design does not allow for causal inference between the identified factors and ITN non-use. The observed associations reflect statistical links without confirming a strict temporal relationship between exposure and outcome. Second, data were partly based on guardian reports, exposing the study to social desirability bias, particularly regarding ITN use; some respondents may have overestimated adherence to health recommendations. Third, the study was conducted within the context of preschool consultations, which may limit the generalizability of the results to the broader pediatric population. Children not attending health services may have different characteristics, including more limited access to preventive interventions. Also, children attending preschool consultations—may introduce a selection bias, as these children are more likely to come from households already engaged with the health system. This could partially explain the high reported utilization rate of ITNs compared to what might be observed in the general population. Fourth, some potential determinants (distance to health facility, actual ITN availability at the time of visit, cultural norms, perception of malaria risk) were not explored in depth. Finally, although multivariate analysis controlled for several confounding factors, unmeasured variables may still influence the observed associations.

## Conclusion

This study highlights a pediatric population from socio-economically vulnerable households, with moderate ITN coverage but excellent use when nets are available. The main barriers identified relate to access to information, ITN availability, and certain structural factors. Independent determinants of ITN non-use include lack of community sensitization, living without a partner, large household size, rudimentary housing, and the rainy season. These results suggest that the issue is not solely one of individual adherence but reflects a broader socio-economic and organizational context. Strengthening community-based interventions, ensuring continuous ITN supply, and adapting strategies to seasonal and structural realities are essential to optimize coverage and contribute to sustainable malaria burden reduction among children.

## Supporting information

S1 File Database (Excel). (XLSX)
